# Tailored approach to sleep health education (TASHE): study protocol for a web-based randomized controlled trial

**DOI:** 10.1186/s13063-016-1701-x

**Published:** 2016-12-08

**Authors:** Natasha J. Williams, Rebecca Robbins, David Rapoport, John P. Allegrante, Alwyn Cohall, Gbenga Ogedgebe, Girardin Jean-Louis

**Affiliations:** 1 Department of Population Health, Division of Health and Behavior, Center for Healthful Behavior Change, NYU Medical Center, New York, NY 10016 USA; 2 Ichan School of Medicine at Mount Sinai, Pulmonary, Critical Care and Sleep Medicine, New York, NY 10029 USA; 3 Department of Health and Behavior Studies, Teachers College, Columbia University, New York, NY 10027 USA; 4 Harlem Health Promotion Center, Columbia University, New York, NY 10032 USA

**Keywords:** Adherence, Sleep health, Blacks, Continuous positive airway pressure, Health education, Health literacy, Internet, Obstructive sleep apnea

## Abstract

**Background:**

Obstructive sleep apnea (OSA) is a sleep disorder that disproportionately affects African Americans (hereafter referred to as blacks). Moreover, blacks may underutilize sleep services including overnight polysomnography. Thus, OSA among blacks may go undiagnosed and untreated, which has significant health consequences, including hypertension, diabetes, cognitive impairment, and daytime sleepiness.

**Design and Methods:**

This two-arm randomized controlled trial will assign 200 participants to a culturally and linguistically tailored web-based sleep educational platform. The website will be developed to ensure that the content is user friendly and that it is readable and acceptable by the target community. Participants will receive login information to a password-protected website and will have access to the website for 2 months. Study assessments will be collected at baseline, 2 months (post-enrollment) and at 6 months (follow-up). We will use qualitative and quantitative methods to develop tailored materials and to ascertain whether tailored materials will increase OSA knowledge and OSA health literacy by comparing blacks exposed to tailored materials versus those exposed to standard sleep health literature. We hypothesize that exposure to tailored OSA information will improve OSA health literacy.

**Discussion:**

Few studies have investigated the racial/ethnic disparities in relation to OSA screening and treatment comparing blacks and whites. Moreover, we know of no interventions designed to increase OSA knowledge and health literacy among blacks. Use of the Internet to disseminate health information is growing in this population. Thus, the Internet may be an effective means to increase OSA health literacy, thereby potentially increasing utilization of sleep-related services in this population.

**Trial registration:**

The study is registered at clinicaltrials.gov, reference number NCT02507089. Registered on 21 July 2015.

## Background

Increasingly, researchers and practitioners recognize that obstructive sleep apnea (OSA) - one of the most commonly diagnosed sleep disorders - disproportionately affects blacks. One of the earliest studies documenting racial/ethnic differences in the rate of OSA compared 225 black and 622 white volunteers, ages 2–86 years, and found that 31% of blacks versus 10% of whites had OSA [1]. More recent studies have also reported similar disparities including adherence to OSA treatment, with blacks using positive airway pressure (PAP) far less than their white counterparts [2–4]. The underlying mechanisms of these disparities are not clear. However, research suggests that the disparities in OSA prevalence may be explained by genetic factors and obesity [5], while disparities in treatment adherence may be partially explained by socioeconomic status [3] and sleep duration [2].

The Institute of Medicine and the National Institutes of Health (NIH) recognize OSA as a chronic disease that requires novel adherence strategies to promote enhanced quality of life and diminish social and economic costs [6]. Yet, our pilot data suggests that blacks tend to underutilize sleep services. This is unfortunate given that there may be a high prevalence of OSA in this community, and that treatment is effective in improving cardiovascular disease (CVD) risk factors, especially blood pressure [7], which disproportionately burden blacks.

### Insufficient sleep and sleep disorders

A recent analysis of data from 12 states conducted by the Centers for Disease Control and Prevention (CDC) illustrated that 35.3% of United States (US) adults reported insufficient sleep and 37.9% reported unintentionally falling asleep during the day [8]. It also appears that there is increased awareness about sleep in the general population. For example, using the National Ambulatory Medical Care Survey, Ford et al. [9] found a 13% increase in the number of physician office visits for sleep complaints and a 200% increase in the number of individuals who reported a sleep disorder diagnosis including OSA, insomnia, and restless leg syndrome. As there appears to be an increase in sleep complaints and diagnoses of sleep disorders, as well as the rapidly growing field of sleep medicine, it is paramount that patients be educated about sleep health, including the importance of screening and adhering to treatment, especially among minority populations who may underutilize sleep services. Blacks as well as other racial/ethnic groups with untreated OSA may be at greater risk for hypertension [10], diabetes [7], cognitive impairment [11], and poor quality of life [12].

### Sleep and health literacy

Health literacy is defined as “the degree to which individuals can obtain, process, and understand basic health information, and services they need to make appropriate health decisions” [13]. Health literacy requires several skills including print literacy (reading and writing), numeracy (recognizing numbers in the case of a prescription), and oral literacy (listening and speaking). It is estimated that 77 million Americans have limited health literacy, costing the healthcare system $106–238 billion a year [14].

Limited health literacy is a major problem in the US, and health literacy is linked to delayed diagnoses, lack of understanding of medical condition, lack of treatment, poor self-management skills, and worse clinical outcomes [15]. Greater rates of limited health literacy are generally reported among minorities, the elderly, and individuals with low educational attainment. A systematic review indicated that patients with low health literacy were generally 1.5 to 3 times more likely to experience poor health outcomes [16].

Patients with limited health literacy may experience difficulty locating providers and services, completing complex health forms, sharing their medical history with providers; they may also be unaware of the link between health risk behavior and poor health outcomes. It is plausible, then, that describing symptoms of sleep disturbance, undergoing screening for sleep disorders, and adhering to treatment, particularly PAP, would require high-level literacy skills. In a cohort of men from Australia who underwent polysomnography, low functional health literacy, another measure of health literacy, was one of the main factors associated with previously undiagnosed OSA (OR = 2.43, 95% CI = 1.40–4.20) compared to men with adequate functional health literacy [17]. These findings also suggest that health literacy is associated with the uptake of OSA screening.

The Centers for Medicare and Medicaid Services requires that the patient use PAP for 4 or more hours per night, 70% of nights in a 30-day period for reimbursement [18]. In addition to the lack of empirical evidence to support this guideline as a necessary and sufficient marker of adherence, this information might be difficult for patients to comprehend; patients may be unable to process the amount of time that they are required to use their PAP mask accurately in order to achieve adherence. In addition, adherence to PAP may require a high level of oral communication skills whereby patients should be able to communicate with their provider whether or not they are experiencing challenges with mask use; the PAP mask may involve several challenges including negative side effects and mask discomfort [19]. It is plausible that some of the adherence issues may be exacerbated when coupled with mental health problems, and there is a high rate of depression among patients diagnosed with OSA. In effect, in an analysis of the 2005–2008 National Health and Nutrition and Examination Survey, Wheaton et al. [20] reported that the rate of depression was two and five times more likely to be reported among men and women, respectively, who were diagnosed with OSA. Thus, adhering to physician recommendations for OSA screening and treatment requires a high degree of health literacy, and there may be significant consequences for those patients with low literacy skills.

Similarly, patients may have difficulty completing commonly used sleep surveys. One study found that 56% of new sleep patients had difficulty completing the Epworth Sleepiness Scale, a commonly used measure for describing sleep symptoms [21]. In another study, 44% of patients with low literacy skills required assistance reading National Sleep Foundation (NSF) and American Academy of Sleep Medicine (AASM) brochures [22]. Using the Rapid Estimate of Adult Literacy in Medicine [23] questionnaire, a survey of patients from 122 sleep centers showed that 16.3% exhibited impaired health literacy [21]. Of note, the Center for Health Care Strategies does not recommend such tools in communities characterized by low literacy levels and trust [24–27], unless these materials are specifically tailored for the intended community [28].

### Objectives

Despite these limited studies, which have significant implications for the field of sleep medicine, we know of no interventions to improve adherence to treatment for sleep disorders or adherence to physician recommendations for sleep screening among patients with low health literacy. Our primary objective is to develop the Tailored Approach to Sleep Health Education (TASHE) program using a multilevel community-engaged approach. We will use themes ﻿from focus group to develop a culturally and linguistically tailored wbsite and video vignettes. We will incorporate those themes into an interactive website with OSA health content designed for lay people. This website will thus serve as a platform facilitating exchange of information between sleep scientists, community health providers, potential patients, and lay health advisors. We will also seek endorsement of TASHE materials by healthcare providers and stakeholders to ensure their adoption and dissemination. Our secondary objective is to evaluate TASHE’s effectiveness in increasing OSA health literacy among blacks. The main hypotheses for this study are: (1) modifiable barriers (e.g., negative beliefs and attitudes toward sleep, perceived bias in healthcare, inadequate access, and lack of cultural and linguistic tailoring of sleep health materials to blacks) will comprise emergent themes from focus groups; (2) participant engagement will increase acceptability of sleep health messages. Stakeholder engagement will facilitate adoption, dissemination, and sustainability of the program. Provider engagement will enable tailoring of messages addressing patient’s concerns about effectiveness, benefits, and harms of treatment options to inform decision-making; and (3) exposure to tailored OSA health messages will improve OSA health literacy.

## Design and methods

This two-arm randomized controlled trial will include use of an interactive, culturally and linguistically tailored website for blacks at risk of OSA. The secondary objective is to increase knowledge about OSA and improve OSA health literacy. The approach was designed based on the formative evaluation that was conducted in the NIH-funded study ‘Metabolic Syndrome Outcome Cohort’ study [29, 30] in which results from focus groups included misperceptions about OSA and mistrust of the healthcare system [31, 32]. Results of that study suggested the need to use an innovative approach to increase OSA knowledge and OSA health literacy via a web-based platform.

## Study design and overview

### A community-engaged approach

**Fig. 1:**
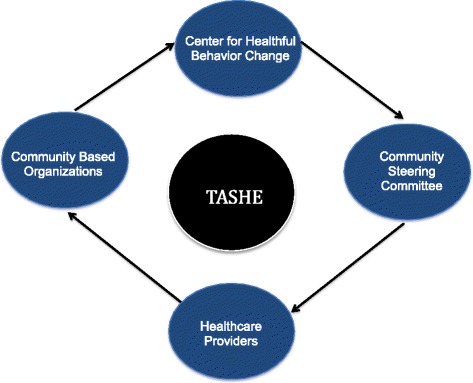
Multi-level Community-engaged approach

We will bring together academic investigators, community providers, and stakeholders, which is considered to be an effective tool to develop interventions, sustainable programs, and policies to address health disparities, and to improve adoption of healthful practices in underserved communities [33, 34]. This partnership approach begins with a research topic of importance to the community (i.e., sleep apnea) and combines knowledge with action to achieve social change in order to improve health outcomes and eliminate health disparities [35]. It is becoming increasingly evident that community engagement in the research process is critical. Doing so leads to identification or refinement of relevant research questions [36], delineates barriers, increases community participation in research [37], improves effectiveness of interventions [38], and enhances dissemination of study results [36] (see Fig. 1).

### Faculty advisory board

We will convene a Faculty Advisory Board (FAB) comprised of the leading sleep medicine physicians, scientists, and researchers, which will be tasked to help develop (review and approval) the sleep health content of all TASHE materials before dissemination. This will ensure that materials emphasize current concepts and understanding regarding sleep health and circadian and sleep biology and promulgate important findings of the National Heart, Lung, and Blood Institute (NHLBI)-funded clinical trials.

### Community steering committee

Our experience conducting minority research has informed us that interventions are best poised to be effective and sustainable when they are culturally appropriate and responsive, and that stakeholders in the partner community endorse them, such that the end result is increased empowerment of community members to address their own health issues. Community academic partnerships, as we have assembled to achieve goals of the TASHE project, have significant advantages over efforts undertaken by researchers alone because such partnerships bring a diversity of expertise that facilitates a more comprehensive understanding of community strengths and needs, as well as improved capacity to plan, execute and evaluate the project. We will create a Community Steering Committee (CSC) [39] bringing together community stakeholders, patients, and health advocates to work with the TASHE project leadership and the FAB. In addition, the CSC will provide feedback on the content of the website to ensure that it reflects the voice of the community and to provide feedback on other programmatic areas including design features and content of the website. The CSC will receive a summary of all research-related findings including themes from the focus groups and preliminary study results throughout the study period.

### Website development

The content of the website will be based on several resources: (1) the FAB and members of the investigative team will advise on the content that should be included and develop all necessary informational sections; (2) a literature review; and (3) a review of existing sleep-related websites and brochures including the AASM, the NHLBI, and the NSF. The FAB will review the content for accuracy and completeness. The content will then be presented to the CSC, which will provide additional suggestions and comments; these will be incorporated into the final content used in the website. The investigative team will review the content to assess health literacy including ease of use, design layout, and readability. We will use the Flesch-Kincaid readability formula and the material will be customized at the fifth-sixth grade reading level.

### Tracking and Internet metrics

The website will be password-protected, accessible only by participants in the exposure arm. By providing a specific password to each participant, we will be able to track their activity on the site using Google Analytics logfile data and relate it to other participant characteristics. Google Analytics is a web analytics service offered by Google that tracks website usage and traffic. We will use this resource to ascertain how participants use the website by linking their login details with metrics generated by Google including pages visited, duration of time on each page, and frequency of visits to the website.

### Role model storytelling development

The primary vehicle by which the project will achieve its goal is through role model storytelling video vignettes. Evidence suggests that video presentation of OSA materials to patients with low literacy skills helped improve OSA knowledge (66% vs. 43%) and usage of continuous PAP (94% vs. 78%) [40]. Video vignettes acquired from interviews with black patients with OSA will demonstrate how poor sleep affects one’s daily functioning and overall health and quality of life; barriers and facilitators to adhering to OSA treatment; undergoing an overnight in-lab and in-home sleep study; and discussing treatment options with a sleep provider. We will use Taylor’s model as the framework guiding the role model story development. According to Taylor’s model [28], role model stories are effective if they reflect realities of individuals from the community to which they are directed. To be credible, the role model will reflect the race, culture, and socioeconomic circumstances of community members. Thus, the appropriate role model will be someone from that community who has adopted recommended healthful practices to be modeled in the story. Thus, initially potential role models recruited from the community will be screened in order to determine if the person could provide enough information about his/her experiences to create a role model story.

### Heurestic testing and tailoring

All role model stories and video vignettes included in the TASHE website will be pretested before randomization. We will conduct heuristic evaluation, led by a member of the investigative team, and cognitive walkthroughs of all web materials, and then perform standard formative usability testing with eight to ten representative participants to ensure the site is well-suited to the needs of the intended community. We will draw upon the work of Kreuter and colleagues [41] that outlines several steps on the way toward the design of tailored materials for health promotion activities. Developing tailored materials is an iterative process whereby participant and population needs are identified, formative materials are designed, feedback from the community or population is obtained, then another round of edits are made before materials can truly be termed ‘tailored’. After the opinions, attitudes, and beliefs about sleep and OSA specific to the population are ascertained, the website will be available for the intervention.

Data obtained from the usability testing include qualitative data obtained from the interviews, as well as quantitative metrics obtained from the computer-tracking software. The data analyst will conduct a qualitative analysis to identify themes in the participant reactions and comments. In addition, the quantitative data collected vis-à-vis the software program will be analyzed using statistical software. Descriptive analysis for the various quantitative metrics will be tabulated to understand the various website components that drew the most attention from participants.

### Focus groups

For aims 1 and 2 of this study, the focus will be on determining barriers preventing uptake of healthful sleep practices in the partner community and developing health messages to promote uptake of these practices. Barriers will be elicited through focus groups, and tailored health messages will be developed and refined. We will conduct five focus groups with patients and community stakeholders. In the first three groups, we will document barriers and factors facilitating or inhibiting uptake of healthful sleep practices. Emergent themes will be presented to the next two groups; here, the primary focus will be on reactions to these themes. Targeted themes will include beliefs, knowledge, values about healthful sleep practices, and mediating or reinforcing factors associated with healthful behavior. We will also conduct focus groups with community stakeholders and with healthcare providers. The purpose of the stakeholder focus group will be on reactions to health messages developed based on themes emanating from focus group with participants in aim 1. During the provider interviews, we will gauge their understanding of participants’ perspectives on benefits and harms of preventive, diagnostic, therapeutic, or sleep healthcare delivery systems. These factors influence participants’ decision-making regarding uptake of healthful practices.

### Eligibility criteria

Participants must meet the following study criteria: inclusion criteria: self-reported race/ethnicity as African American, African, Caribbean American or black men and women; ages ≥18 years; accessible by telephone; no plans to move away from the region within the year following enrollment; consent to participate, which includes permission to release medical record information; documented OSA risk based on scores received from the Apnea Risk Evaluation System (ARES) questionnaire [42]. Internet access is not an eligibility criterion, as participants will receive an iPad with cellular service.

Exclusion criteria will include: progressive medical illness in which disability or death is expected within 1 year; impaired cognitive or functional ability which would preclude meaningful participation in the study; sleep apnea diagnosis; stated intention to move within the same year of enrollment.

### Recruitment

We will use several recruitment strategies. First, we will utilize a recruitment funnel that we have developed from several other Institutional Review Board (IRB)-approved NIH-funded studies related to sleep health. Participants will be contacted via phone to ascertain their level of interest. If participants express interest, they will be invited to come to our office at New York University. Then, a member of the research team will provide detailed information about the study protocol, and read the consent form. Participants will have time to ask questions.

Second, we will host information sessions with prospective participants. These sessions will last approximately 20 minutes and will be led by a senior member of the investigative team. The purpose of the session is to (1) provide detailed information about the study protocol, (2) discuss requirements and expectations of participants, and (3) ascertain interest in moving forward with the study. Participants will receive information materials and a consent form that they may take home to discuss with their family, friends, or doctor, before they agree to sign the consent form. This will allow participants enough time to make an informed decision about whether or not to participate in the study. Participants will have time to answer questions of the investigative team.

Third, we will rely on established relationships with community-based and faith-based organizations including barbershops and churches. Through these efforts, the research team is able to go on site to gauge participant interests. We will follow the same procedures throughout the study, which involve: (1) providing detailed information about the study, (2) providing study materials for participants to review on their own time, and (3) allowing participants enough time to ask questions freely.

Finally, recruitment materials including flyers, brochures, and fact sheets will be developed in conjunction with the CSC. These materials will be at the fifth-sixth grade reading level. All materials provide an overview of what is involved in participating in the study, and will be placed in community venues including barbershops, community-based organizations, and churches. The CSC members will provide feedback to ensure that the materials are clearly written, and that they are acceptable to the community. The Project Manager in consultation with the Principal Investigator (PI) will oversee recruitment and consent.

### Allocation

**Fig. 2:**
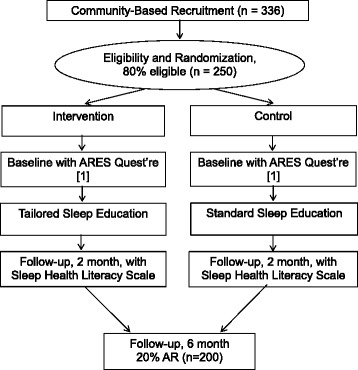
CONSORT flow diagram

Individuals will be eligible for randomization once their baseline questionnaire is completed. Randomization will be carried out in pairs using a table of random permutations conducted by the Program Manager. One member of each pair will be assigned to the Internet-based group and the other to the control group, using sealed envelopes. This strategy ensures equal allocation to the two groups, and that both have similar characteristics regarding measured and unmeasured background variables (i.e., patient characteristics and lifestyle behavior at baseline, medical comorbidities, and situational/contextual factors), which could potentially affect study outcomes. The Program Manager will hold the sequence of assignments and will dispense the code for each new patient upon request by the Research Assistant (RA). If a participant requests to be withdrawn from the study, the Program Manager will assign the code for the next new patient. The Program Manager  will maintain a record of who was randomized to which group to maintain integrity of planned analysis. Due to the nature of the study, the Program Manager will not be blind to the study arms. Randomization will be ongoing until sample size goals are reached: 100 per study arm and in accordance with the CONSORT randomization guidelines as shown in Fig. 2.

### Measures

Screening of baseline measures: we estimate that completion of the baseline study measures will take up to 15 minutes. For participants who are eligible and enrolled into the study, we estimate that the additional screening questionnaires will take up to 30 minutes.

#### Sociodemographic variables

Sociodemographic variables will include age, type of health insurance, marital status, education, number of children living in household, number of adults living in household, employment status, and annual household income.

#### Apnea Risk Evaluation System questionnaire

The ARES includes questions on sleep patterns, daytime functioning, knowledge of sleep apnea, and diseases associated with sleep apnea [that is, hypertension (HTN), diabetes mellitus (DM), heart disease] [42].

#### Epworth Sleepiness Scale

The Epworth uses a Likert-type scale in which the respondent indicates the most appropriate number ranging from 0 = would never doze or sleep to 3 = high chance of dozing or sleeping based on a given situation. A score of 10 or more is considered excessive sleepiness [43].

#### Apnea Knowledge Test (AKT) [44] and Apnea Beliefs Scale (ABS) [44]

These two questionnaires measure participants’ understanding and beliefs of obstructive sleep apnea and CPAP treatment. The AKT includes 13 questions with a yes/no response choice and two open-ended questions; the ABS includes 24 items on a five-point Likert scale ranging from “strongly agree” to “strongly disagree”. A higher score on the AKT indicates better knowledge and understanding of the illness and treatment and a higher score on the ABS indicates greater likelihood of adherence to treatment [44].

#### Change Assessment Scale (CAS)

The CAS is a 32-item instrument that assesses a person’s readiness to change. Participants evaluate the extent to which they strongly agree or strongly disagree with various statements such as, “I’ve been thinking that I might want to change something about myself” and “I have problems and I really think I should work on them.”

#### Medical history

Medical history includes family history of sleep disorders, obesity, DM, HTN, dyslipidemia, and CVD; history of other preventive behaviors (for example, prostate examinations), and other health behaviors (for example, regularity of physical examinations, cholesterol screening, smoking and drinking habits, and exercise habits) [45].

#### Sleep Hygiene Index (SHI)

The SHI is a 13-item instrument that measures sleep hygiene behaviors. It was derived from the diagnostic criteria for inadequate sleep based on the International Classification of Sleep Disorders. Participants are asked about the frequency that they engage in specific behaviors with a response of “always,” “frequently,” “sometimes,” “rarely,” or “never.” Behaviors such as using the bed for things other than sleeping and sex, and daytime naps longer than 2 hours are included [46].

#### Short Test of Functional Health Literacy in Adults (S-TOFHLA)

The S-TOFHLA is a measure of a patients reading and comprehension of both print and numeracy health-related information [23].

#### Sleep Apnea Literacy Scale

This is a 24-item instrument designed to measure patient-level knowledge, efficacy, and literacy barriers to effective treatment for sleep apnea. Specific items include statements such as “when breathing stops during sleep this is called sleep apnea” and “poor concentration can be caused by sleep apnea.” Participants are asked to mark their confidence for these statements about sleep apnea on a scale from “very confident this is true” to “not at all confident this is true” [47].

#### Internet Self-Efficacy Scale

This scale will measure confidence in the ability to use the Internet. Participants rate their confidence on a Likert-type scale and are asked statements such as, “using the Internet to gather data” and “turning to an online discussion group when help is needed” [48].

A complete list of measures and the data collection schedule is provided in Table 1.

**Table 1:** Study measures and schedule to acquire data

	Time point		
Measures	Baseline	2 months	6 months
Demographic and clinical variables	X		
Medical Outcomes Study Short Form 36	X		
Self-Efficacy Scale	X	X	X
Change Assessment Scale	X	X	X
Intrinsic Motivation	X	X	X
Apnea Risk Evaluation System Questionnaire	X		
Sleep Hygiene Index	X	X	X
Apnea Knowledge/Apnea Belief Scale	X	X	X
Sleep Apnea Literacy Scale	X	X	X
Test of Functional Health Literacy in Adults (short version)	X	X	X
Internet Self-Efficacy Scale	X	X	X

### Intervention

Participants randomized to the intervention arm will receive an iPad with free access to a password-protected interactive culturally and linguistically tailored sleep informational website. Study assessments will be collected at baseline, 2 months (post-enrollment) and at 6 months (follow-up). Participants will receive a brief 10-minute tutorial by the study team on login procedures and use of the website. Participants will also be able to call the project staff to troubleshoot any issues with the website. Given that this is a web-based intervention, the study setting is the participants’ home.

### Control condition

Participants randomized to the control condition will receive an iPad ﻿with﻿ free access to the NSF and NHLBI website via an iPad. Currently, there is no specific comparative effectiveness data evidencing superiority of any website or other format for increasing sleep health literacy. Furthermore, there has been previous research to suggest the overall difficulty that patients experience in using the NSF materials. Thus, our study will ascertain effectiveness of a tailored website compared to the standard websites. Participants in the control condition will have access to the culturally and linguistically tailored sleep informational website at 6 months post-enrollment date.

## Design and methods: data collection, management, and analysis

Survey data will be collected through a password-protected website, REDCap, a secure website for managing and collecting online survey da﻿ta, which meets the standards set forth by the NYULMC IRB and HIPPA offices. Additional information about data entry, storage, quality and can be found in the IRB study protocol.

### Plans to promote retention

Participants in the intervention arm will be contacted weekly by the PM to ensure ongoing use of the website and access to intervention materials. We will track participant usage based on Google Analytics.

### Sample size

Guided by principles governing the use of focus group data, the sample size will be determined when we reach a point of “saturation”, which is when additional group sessions are not likely to produce new themes. Based on previous research [49, 50], we expect that five groups will be sufficient to capture key themes for the project. In the secondary objective - assuming a medium effect size (d = 0.32), as derived from Cohen [51], and a sample size of 200 - the study will be adequately powered (>85%) to detect significant differences between participants likely to show improvement in health literacy scores and those who are not using Fisher’s exact tests (critical X^2^ = 7.98). In a previous pilot study, we found that 40% of black patients in the community adopted recommendations for OSA treatment after exposure to health messages, which compares to 27% who did without such exposure. Assuming a 13% increase in the likelihood of adoption of health messages and a sample size of 200, the proposed study will have adequate power for a preliminary test of the hypothesis that exposure to health messages will foster improvement in sleep health literacy. Achieving the goal of a 13% increase would have an important public health impact. A two-tailed test, with 85% power and α = 0.05 comparing 27% to 40% requires 100 participants per arm [52].

### Statistical analysis

Zemke and Kramlinger’s procedures will be used to analyze qualitative data for aims 1 and 2. Focus groups will be recorded and transcribed verbatim. Transcripts will be reviewed line by line and assigned codes or labels to develop themes. Transcripts will be coded and reviewed by two independently trained RAs and the study team will review transcripts in case of any disagreement with codes and themes. For the qualitative data our first hypothesis is that modifiable barriers (e.g., negative beliefs and attitudes toward sleep, perceived bias in healthcare, inadequate access, and lack of cultural and linguistic tailoring of sleep health messages to minorities) will be consistent themes in focus groups. For hypothesis 2: participant engagement will increase acceptability of health messages depicted in posters and dispensed through the interactive website. Stakeholder engagement will facilitate adoption, dissemination, and sustainability of the program. Provider engagement will enable tailoring of health messages addressing participant’s concerns about effectiveness and benefits of different treatment options to inform decision-making. Since we plan to analyze both qualitative and quantitative data, we’ll adhere to Office of Behavioral and Social Sciences Research (OBSSR)-recommended ‘Best Practices for Mixed Methods Research in the Health Sciences’.

For aim 3, we will examine which subjective measures are associated with increased OSA health literacy using multivariate logistic regressions. The main dependent measure will be a binary factor: [yes versus no]; candidate predictors are indicated in Table 1. We will use multivariate analysis to explore whether sociodemographic variables (e.g., age, income, education), lifestyle practices (e.g., diet, exercise, smoking, and drinking habits), Internet self-efficacy, and baseline sleep measures (e.g., OSA knowledge, OSA beliefs, and knowledge of sleep hygiene) indexing OSA health literacy improved after exposure to health messages. We assess OSA health literacy with the Sleep Apnea Literacy scale, a 24 -item questionnaire developed by a sleep expert, and expert in scale design. The questionnaire assesses sleep apnea knowledge and sleep apnea clinical management. Sample items include “When breathing stops during sleep this is called sleep apnea” and “Loud snoring can be a sign of sleep apnea.” Ninety-one participants average age 38, and 48% white, 27% African American completed the questionnaire. The questionnaire was developed using exploratory and confirmatory analyses. The questionnaire includes items across three scales, including sleep apnea literacy (component alpha 0.74), sleep apnea self-efficacy (component alpha 0.76), and sleep apnea clinical management (component alpha 0.65). The concurrent scale validity is 0.85. To compare participants who exhibited improvement in health literacy and those who did not 2 months post-enrollment, *t* tests and chi-squared tests will be used. Multivariate analysis of covariance will be used to assess effects of sleep health messages on sleep measures (e.g., OSA knowledge, OSA beliefs, knowledge of sleep hygiene, and S-TOFHLA scores); effects of confounders will be adjusted. Baseline subjective factors will be entered in a linear regression model in order to determine which factors are associated with higher health literacy scores. In post hoc analyses, we will also explore whether combined effects of exposure to tailored web-based content, and video vignettes received through the website had greater impact on improvement in sleep apnea literacy scores.

Both selection and attrition biases might occur during data acquisition for aim 3. Selection bias might result from participant’s refusal to participate and attrition bias could result in an unbalanced design. Appropriate statistical techniques (e.g., Heckman two-stage approach GEE probit model, and Monte Carlo [53] will be used to address such biases, or problems that might result from missing data. Adequate statistical corrections will be made in final statistical analyses to increase generalizability of the findings. Sociodemographic data obtained from those who did not participate will be used to determine whether they are similar to those in the study [54]. Likewise, baseline characteristics of participants who withdrew from the study will be contrasted with completers to determine whether any attrition bias may have occurred.

The Biostatistician will manage study data under the PI’s supervision. He will create computerized data collection forms to ensure integrity and accuracy of data entry. He will help to minimize the problem of missing data by resolving omissions and errors as they arise. Throughout the study, the PI will oversee data entry and verification, ensure adherence to coding, and clean and edit protocols. Participant-identifying information will be kept in a locked file cabinet with only numerical identifiers in computer files.

## Data monitoring

In compliance with NIH requirements, we will establish a data safety and monitoring plan (DSMP). The purpose of the DSMP is to ensure the safety of participants and the validity and integrity of the data. In adherence to NIH policy regarding data sharing, the DSMP will be tasked to develop its data-sharing plan in consultation with an external representative. Data will be made available from the institutions’ website once the main findings of the project have been published. The plan will incorporate language to ensure that the rights and privacy of people who participate in NIH-sponsored research are protected at all times. Thus, data will be free of identifiers that would permit linkages to individual research participants and variables that could lead to deductive disclosure of individual participants. The DSMP internal committee will review all requests for data before access to the data is granted.

## Discussion

OSA is one of the most commonly diagnosed sleep disorders that disproportionately affect blacks. With the rapidly changing field of sleep medicine, including the use of home-based sleep studies, patients may require assistance navigating sleep health services [55]. In addition, there are various treatment options, although CPAP is the most effective. Moreover, treatment for OSA is efficacious and can reduce symptoms of co-morbid health conditions including CVD, diabetes, and hypertension. Unfortunately, adherence to treatment for OSA remains suboptimal and blacks in particular may use CPAP far less than their white counterparts, thus limiting their ability to benefit from treatment. Although several factors have been proposed as contributing to poor adherence, including machine difficulties and psychosocial factors, few studies have explored the role of health literacy [19, 55]. Limited health literacy is associated with several health outcomes and requires a complex set of skills. Thus, it is plausible that individuals with limited OSA health literacy would benefit from the intervention, which will utilize a web-based approach to increase OSA health literacy that we are testing.

In conclusion, there is growing interest in using telehealth among minorities [56–58]. Studies are beginning to show that Internet use in minority communities has increased over the last several years. A random-digit telephone survey of New York residents found that 77% had computer access and 65% used the Internet [59]. Online information affects health habits of New York minorities, with 47% in a telephone survey stating it changed their exercise routines and 44%, their eating habits [60]. Computer-based pre-screening is well accepted for HIV testing, with 80% of respondents in one study preferring the computer-based screening compared to other methods [61]. Thus, we believe that an approach that builds on what is now known about the potential role of web-based information might be an effective means to increase OSA knowledge and OSA health literacy, thereby increasing utilization of sleep-related services among blacks.

### Trial status

Recruiting.

## Abbreviations

AASM: American Academy of Sleep Medicine
ABS: Apnea Beliefs Scale
AKT: Apnea Knowledge Test
ARES: Apnea Risk Evaluation System
PAP: Positive airway pressure
CSC: Community Steering Committee
CVD: cardiovascular disease
DM: diabetes mellitus
DSMP: data safety and monitoring plan
FAB: Faculty Advisory Board
HTN: hypertension
IRB: Institutional Review Board
NHLBI: National Heart, Lung, and Blood Institute
NIH: National Institute of Health
NSF: National Sleep Foundation
OSA: Obstructive Sleep Apnea
PI: Principal Investigator
S-TOFHLA: Short Test of Functional Health Literacy in Adults
RA: Research Assistant
TASHE: Tailored Approach to Sleep Health Education
